# Exploring the Efficacy of Virtual Reality Training in Obstetric Procedures and Patient Care—A Systematic Review

**DOI:** 10.3390/healthcare13070784

**Published:** 2025-04-01

**Authors:** Ioana Gabriela Visan, Cristian Valentin Toma, Razvan Petca, George E. D. Petrescu, Aniela-Roxana Noditi, Aida Petca

**Affiliations:** 1 Department of Obstetrics and Gynecology, ‘Carol Davila’ University of Medicine and Pharmacy, 020021 Bucharest, Romania; ioana.visan@umfcd.ro (I.G.V.); aida.petca@umfcd.ro (A.P.); 2 Department of Urology, ‘Carol Davila’ University of Medicine and Pharmacy, 020021 Bucharest, Romania; 3 Department of Urology, ‘Prof. Dr. Th. Burghele’ Clinical Hospital, 050659 Bucharest, Romania; 4 Department of Neurosurgery, ‘Carol Davila’ University of Medicine and Pharmacy, 020021 Bucharest, Romania; george.petrescu@umfcd.ro; 5 Department of Neurosurgery, Bagdasar-Arseni Clinical Emergency Hospital, 041915 Bucharest, Romania; 6 Department of Surgical Oncology, ‘Carol Davila’ University of Medicine and Pharmacy, 020021 Bucharest, Romania; aniela.noditi@umfcd.ro; 7 Department of Surgical Oncology, Institute of Oncology ‘Prof. Dr. Alexandru Trestioreanu’, 022328 Bucharest, Romania; 8 Department of Obstetrics and Gynecology, Elias Emergency University Hospital, 011461 Bucharest, Romania

**Keywords:** obstetrics and gynecology, virtual reality, medical simulation, medical training, pacient care

## Abstract

Background: As technology continues to shape society, younger generations are increasingly accustomed to its integration into daily life, making it mandatory for medical educators to adopt innovative tools like virtual reality (VR). This systematic review examines the efficacy of VR in obstetric training and patient care, focusing on its impact on educational engagement, procedural skill acquisition, and pain management in obstetric patients. Methods: A systematic review of the current literature was conducted using databases: PubMed, Web of Science, Science Direct, Scopus, Embase, The Cochrane Library, and Clinicaltrials.gov analyzing randomized controlled studies on VR’s use in obstetric training and patient care. Inclusion criteria focused on studies evaluating VR’s role in enhancing clinical skills, and pain and anxiety management during labor and procedures. Only randomized controlled trials published in English were considered. The risk of bias was assessed using RoB 2 for RCTs. Data extraction and quality appraisal were performed independently by two reviewers. Results: A total of 18 studies met the inclusion criteria. Among them, 13 studies focused on VR for pain relief and anxiety reduction, and 5 studies on medical training and skill acquisition. Most studies used immersive VR headsets, while some utilized interactive VR or serious gaming platforms. Adverse effects such as motion sickness and visual discomfort were reported in a few cases but did not significantly impact participant engagement. Conclusions: VR holds the potential to improve obstetric training and patient care by aligning with the learning preferences of younger generations and enhancing both educational and patient care experiences. However, heterogeneity in sample sizes, participants, and intervention types limits generalizability. Further large-scale, high-quality RCTs are needed to validate findings and standardize VR applications in obstetrics. This review was registered in PROSPERO (Registration ID: CRD42024619197).

## 1. Introduction

Virtual Reality (VR) is an immersive technology that integrates computer, multimedia, graphics, and simulation technologies to create a virtual environment. Through specialized equipment, users experience various sensory inputs such as sight, sound, and sometimes even smell, fostering an immersive feeling. Within this virtual realm, users can engage with objects and environments, enabling interactive experiences [1].

VR technology comprises two main components: hardware and software. The hardware includes devices like the head-mounted display, or VR headset, equipped with sensors to track users’ head movements and adjust visual output accordingly. On the other hand, VR software generates three-dimensional (3D) environments that users interact with. These environments range from entirely fictional settings found in entertainment media like videos and games to practical applications such as VR-based education and training simulations [2].

VR technology is categorized into three main types: immersive, semi-immersive, and non-immersive. Immersive VR integrates elements from the real world into the virtual environment to enhance users’ sensory experiences within the simulated setting. In semi-immersive VR, users can interact with their surrounding physical environment while engaging with the virtual content, leading to partial engagement with the virtual world. On the other hand, non-immersive VR involves computer-generated simulations displayed on desktop screens, with users interacting through peripheral devices like a mouse or joystick [3]. These distinctions in VR technology play crucial roles in medical applications, influencing the depth of immersion and user interaction in various healthcare contexts [4].

The development of VR in medicine has been closely linked to the advancements made in consumer-grade VR technology. Starting from 2011, companies such as Valve, NVIDIA, and Oculus played a pivotal role in transitioning head-mounted display (HMD)-based VR from specialized laboratory settings to mainstream accessibility, which facilitated its adoption in medical training and patient care [4].

Unlike traditional methods, VR allows medical students to practice techniques without involving real patients, enabling them to familiarize themselves with various situations and refine their skills through unlimited repetitions. VR simulators offer realistic feedback, enabling self-assessment and providing professional guidance to enhance technique. Moreover, they can simulate surgical complications, preparing students for real-world challenges, and teachers can utilize VR to demonstrate intricate procedures, facilitating deeper understanding [5].

The integration of VR in medical procedures has seen important advancements in more recent years, transforming both the training of medical professionals and patient care. VR is being increasingly used to simulate complex medical scenarios, allowing healthcare professionals to practice and improve their skills in a more controlled environment. Studies have shown that VR-based simulations enhance the learning outcomes of medical professionals, particularly in high-risk specialties such as surgery and obstetrics, by offering these immersive, realistic training experiences [6]. This technology not only helps to improve the technical skills of physicians but also provides patients with therapeutic interventions, enhancing their overall healthcare experience [7].

Unlike traditional methods, VR allows medical students to practice techniques without involving real patients, enabling them to familiarize themselves with various situations and refine their skills through unlimited repetitions. VR simulators offer realistic feedback, enabling self-assessment and providing professional guidance to enhance technique. Moreover, they can simulate surgical complications, preparing students for real-world challenges, and teachers can utilize VR to demonstrate intricate procedures, facilitating deeper understanding [8].

From a patient’s perspective, VR technology can offer several benefits. Immersive simulations help patients better understand not only medical conditions but also treatment plans and along with this, reduce anxiety [7]. VR also allows patients to experience procedures virtually, increasing preparedness and control [8]. During rehabilitation, VR makes therapy more engaging and enjoyable, enhancing motivation and treatment adherence, which ultimately leads to better outcomes and improved overall healthcare experience [9].

VR models in healthcare can serve as powerful tools for enhancing medical education, skill acquisition, decision-making, and patient management, with studies demonstrating their effectiveness [7]. These models can improve anatomical comprehension, reduce surgical errors by up to 53.7%, facilitate intensive care unit (ICU) training, and even alleviate patient anxiety through immersive therapeutic interventions [7,10,11,12]. Given these applications, we aimed to explore whether similar benefits extend to obstetrics by systematically reviewing the existing literature on VR applications in this field.

While the integration of VR technology in medical education has garnered interest, significant gaps remain in understanding its full potential in obstetric training and patient care. This systematic review aims to investigate these gaps by examining how VR can change traditional obstetric training and its implications for improving patient outcomes and educational engagement.

## 2. Materials and Methods

### 2.1. Search Strategy

A systematic literature search was conducted from September 2024 until December 2024 to explore the efficacy of VR training in obstetric procedures and its implications for healthcare professionals and patient care. The search was carried out using databases: PubMed, Web of Science, Science Direct, Scopus, Embase, The Cochrane Library, and Clinicaltrials.gov. The timeframe for the search was from 1 January 2002 to 12 December 2024, reflecting the rapid advancements in VR technology over the past two decades. This systematic review was conducted following the Preferred Reporting Items for Systematic Reviews and Meta-Analyses (PRISMA) guidelines, following the PRISMA checklist to ensure transparency and rigor in reporting.

Keywords used in the search included combinations of terms such as “virtual reality” OR “VR”; “obstetric procedures” OR “obstetrics training” “healthcare professionals” OR “medical students” OR “midwives” OR “obstetricians” OR “nurses” “caesarean section” OR “C-section” “labor management” OR “childbirth” “medical education” OR “training” “patient care” OR “patient outcomes” “VR simulation” OR “virtual simulation “ together with Boolean combinations.

### 2.2. Inclusion and Exclusion Criteria

The inclusion and exclusion criteria used in this study are summarized in Table 1.

### 2.3. Screening and Selection Process

The study protocol was registered by the International Prospective Register of Systematic Reviews under the ID number CRD42024619197. The initial search was done by two independent reviewers (IGV and CVT), and it identified a total of 1221 records from the databases. After removing duplicate records, 1176 unique records were screened. Following the screening process, 1122 records were excluded based on the title and abstract, which did not align with the inclusion criteria. Of the remaining 54 reports sought for retrieval, 4 were not retrieved due to accessibility issues, leaving 50 reports assessed for eligibility. After a thorough eligibility assessment, 32 reports were excluded, resulting in 18 studies included in the final review. Where conflicts arose that could not be resolved by consensus discussion alone, a third reviewer acted as an independent adjudicator (AP).

**Figure 1:**
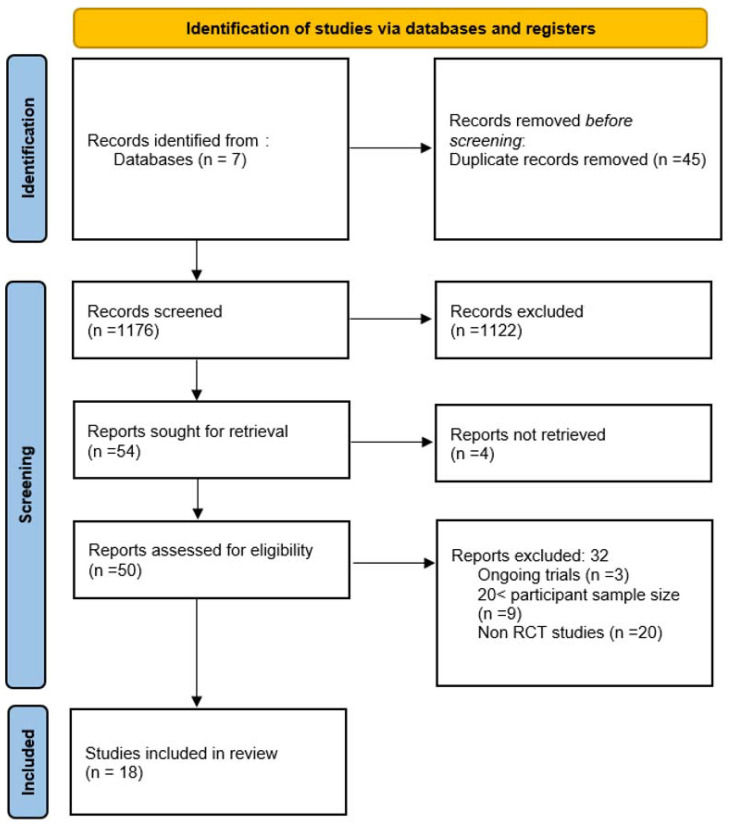
Prisma flow diagram depicting the screening process [13].

The screening and selection process done independently by two reviewers is depicted in the PRISMA flow diagram (Figure 1).

### 2.4. Risk of Bias Assessment

The risk of bias for the included studies (Table 2) was assessed independently by two reviewers (GEDP and RP) using the RoB 2: A revised Cochrane risk-of-bias tool for randomized trials [14]. This tool evaluates the methodological quality of randomized controlled trials across five specific domains of potential bias. For this review, we systematically documented the evaluation for each study under the following domains, bias arising from the randomization process, bias due to deviations from intended interventions, bias due to missing outcome data, bias in measurement of the outcome, bias in selection of the reported result. The overall bias consists of a measure of low, high, and some concerns regarding the studies analyzed. 

While no studies included in this review were at high risk of bias, we have identified some concerns in certain domains. As shown in Table 2, the most frequently observed concerns were related to deviations from intended interventions and measurement of outcomes. This is relevant in VR-based interventions, where full blinding is inherently difficult, as participants are always aware of the intervention they are being subjected to. Studies such as Jeong et al. [23] and Gür et al. [30] had concerns regarding deviations from the intended intervention, which could introduce performance bias. Similarly, we have identified in a study by Akin et al. [29] some concerns in outcome measurement, potentially impacting the reliability of reported results. However, these biases were not severe enough to classify the studies as high-risk, and we tried to mitigate their influence by including studies with larger sample sizes and rigorous methodological designs. Given these factors, while some concerns exist, the overall risk of bias remains low, supporting the validity of our findings.

### 2.5. Data Extraction and Synthesis

In Table 3 and Table 4, we have listed the studies included in our review. They are split into two categories: obstetrical training and pain and anxiety management. Because of the heterogeneity of study outcomes, participants, and type of VR used, we decided to go with a narrative synthesis approach. Although all the studies included are RTC to add strength to our review, we did include RTC with a smaller sample size (*n*- 34 participants) to better understand the role of using virtual reality in training obstetrical procedures because of scarce literature regarding training in obstetrics using VR tools. To better understand the studies focused on obstetrical training, Table 5 lists the types of VR used, the educational tools they were compared with, and the evaluation methods used.

## 3. Results

Of a total of 18 studies included in our review, different outcomes were measured. The use of VR was primarily used in a teaching context for training in high-risk obstetrical procedures and for improving anxiety and pain levels in obstetrical patients.

Below are the tables (Table 6, Table 7 and Table 8) summarizing the outcomes of studies based on the narrative synthesis.

### 3.1. Pain Relief and Anxiety Management

Despite the use of VR technology aimed at relieving pain and reducing anxiety levels for obstetrical patients, the anticipated consistent reduction in pain and anxiety scores during obstetric procedures has not been observed across the studies reviewed [20,21,22,24,28].

A randomized controlled trial published in 2021 evaluated the efficacy of VR as a distraction technique for managing acute pain and anxiety during mid-trimester amniocentesis. A lot of 60 women were randomly assigned to either a VR intervention group or a standard care group. Pain levels were measured using the visual analog scale (VAS), while anxiety was assessed with Spielberger’s state-trait anxiety inventory (STAI). The study found that the VR group experienced significantly less pain (VAS score 2.5 ± 1.5) compared to the standard care group (VAS score 3.8 ± 1.7) (*p* = 0.003). They found no significant differences in anxiety levels between the two groups [28].

A clinical pilot study assessed the feasibility of using VR to reduce pain during the external cephalic version (ECV), a procedure that causes moderate pain. The trial involved 50 women, randomly assigned to either a VR group or a standard care group (*n* = 25). The VR group used a headset to view “Sky Lights”, an immersive experience involving lighting Chinese lanterns in a peaceful night sky setting, with head tracking and audio stimulation. Pain, anxiety, vital signs, and user experience were measured before and after the procedure. Results showed no significant differences in pain scores, ECV success rates, or anxiety levels between the VR and standard care groups. However, women in the VR group anticipated more pain pre-procedurally. Despite this, 80% of the VR group would use VR again, and 88% would recommend it to others. The study concluded that VR is a feasible and safe method for use during ECV, providing a basis for future research [31].

A study aimed to compare the effectiveness of VR and chewing mint gum in reducing childbirth pain and anxiety concluded that both methods reduce pain and anxiety during the first stage of childbirth. This single-blind, clinical trial involved 93 mothers divided into three groups: chewing gum, VR, and control. Both pain and anxiety scores were significantly lower immediately and 30 min after the intervention in the VR and chewing gum groups compared to the control group [26].

In a 2022 study focusing on reducing labor pain using VR, results were also promising. Conducted as a double-blind randomized controlled trial, it involved 273 pregnant women at a maternity hospital in eastern Anatolia, Turkey. The participants were divided into five groups, each receiving different VR interventions: videos of newborn photographs with classical music, a newborn photograph album, an introductory film of Turkey, only classical music, and routine hospital care. The results showed that all VR interventions significantly reduced labor pain compared to routine care. Specifically, the interventions featuring videos of newborn photographs with classical music and the newborn photograph album were the most effective [30].

In another recent study evaluating the effectiveness of immersive VR on patient satisfaction and pain relief among women in labor, results showed elevated levels of satisfaction and improved pain scores. Conducted as an RCT, 42 laboring women were allocated to either VR intervention or control groups. The results showed high levels of patient satisfaction with immersive VR, with a mean satisfaction score of 87.7 ± 12.9 out of 100. Notably, 95% of women in the VR group expressed willingness to use VR in future labor. Additionally, VR significantly improved pain scores, with mean pain scores decreasing from 2.6 ± 1.2 pre-VR to 2.0 ± 1.3 post-VR (*p* < 0.01) [24].

Although the results of studies investigating pain and anxiety reduction during various pain-provoking procedures such as amniocentesis have yielded contradictory findings, those focused on reducing pain and anxiety in labor seem to suggest a consistent outcome [24,26,28,30]. VR also showed efficacy in reducing pain perception during labor and in episiotomy repair studies likely due to the immersive distraction provided by VR [20,21,22,24].

When introducing VR into the operating room was explored, patients were less stressed, and maternal satisfaction was high. In a randomized controlled clinical trial conducted in Saudi Arabia, researchers investigated the impact of VR on anxiety, stress, and hemodynamic parameters during cesarean section. The study involved 351 low-risk pregnant women undergoing elective CS with regional anesthesia. The VR group, exposed to calming 3D natural videos with Quranic recitation or music via VR glasses, demonstrated significantly lower stress and anxiety levels immediately after skin closure and 2 h postoperatively compared to the control group [25].

### 3.2. Training Outcomes

Training-focused studies consistently demonstrated that VR enhances knowledge retention, psychomotor skills, and confidence among healthcare professionals. Both immersive VR and serious game-based VR approaches were used.

Immersive VR simulation training for CS has been found to effectively improve healthcare professionals’ knowledge and confidence in managing obstetric situations, such as premature rupture of membranes, and performing CS procedures [18]. In a recent randomized controlled trial involving 105 participants, the VR group (*n* = 53) received VR simulation training, while the control group (*n* = 52) watched a video presentation. Both groups completed pre- and post-intervention questionnaires and a mini-test quiz. The results showed that the VR group had significantly higher confidence and knowledge scores in managing PROM and performing CS compared to the control group, indicating that VR simulation is an effective tool for enhancing medical training in these areas [18].

VR offers an interactive and realistic environment for practicing obstetric maneuvers, as demonstrated in a study where a 360°-VR scenario was developed to simulate obstetrical maneuvers required to manage shoulder dystocia [17]. This study evaluated the effectiveness of a virtual reality training program for managing shoulder dystocia (SD) compared to traditional theoretical training. Using a prospective, case-control, single-blind, randomized crossover design, participants initially received either VR training via a 360° video or a theoretical briefing. Both groups then performed manikin-based training and were assessed on human skills (HuFSHI), adherence to the HELP-RER algorithm, and task load (TLX). After 12 weeks, the groups switched training methods. Results showed that the VR-trained group had significantly better HELP-RER scores, faster diagnosis-to-delivery times, and lower task-load scores compared to their initial theoretical training session [17].

In a single-center randomized controlled trial, medical students (*n* = 35) engaged with virtual reality to learn about fetal lies and presentation, while a control group (*n* = 34) utilized traditional 2-dimensional images. The virtual reality learning environment (VRLE), delivered via a mounted display headset, facilitated an immersive exploration of fetal positioning. Although results showed a non-significant trend towards improved knowledge outcomes in the VRLE group compared to traditional methods, 70% of VRLE participants correctly identified fetal positioning, compared to 56% in the control group. Students in the VRLE group completed tasks more efficiently and expressed higher satisfaction and confidence levels with the learning experience [27].

In a study investigating the impact of VR in maternity nursing education, comparing its effectiveness to traditional simulation-based training, results show increased knowledge and confidence levels in the VR group. This RTC included nursing students (*n* = 59) assigned to either a VR or conventional simulation group. The primary outcomes assessed included knowledge acquisition, self-efficacy, and confidence in maternity nursing skills. Results demonstrated that the nursing students in the VR group scored significantly higher in the post-intervention knowledge assessments compared to those in the control group (*p* < 0.05). Additionally, the VR group showed increased self-efficacy and confidence levels in managing vaginal births [23].

### 3.3. Types of Virtual Reality Used and Side-Effects

As seen in Table 9, the preferred VR used in obstetrics seems to be immersive virtual reality, using headsets.

Although we can see that immersive VR is broadly used for psychomotor, and procedural training and as a distraction technique to lower anxiety and pain in obstetrical patients, it does not come without side effects.

Motion sickness or discomfort was reported in studies where a few participants experienced dizziness or disorientation during prolonged use [15,24]. This phenomenon, often referred to as “VR sickness”, arises when visual stimuli are not well synchronized with head movements [33]. Eye strain and fatigue were noted in studies, particularly after extended focus on VR screens, which can cause temporary visual discomfort, especially for participants unfamiliar with the technology [19,29].

Cognitive overload was identified in a study where some trainees reported feeling overwhelmed by the immersive environment, especially when engaging in complex tasks such as CS simulations [18]. This may result from the intense sensory input characteristic of immersive VR. Mild anxiety or resistance was observed where a small group of trainees expressed hesitation or stress, particularly those unfamiliar with VR technology [17]. Limited physical discomfort was reported where women in labor mentioned mild discomfort from wearing headsets during contractions, highlighting ergonomic challenges during active physical processes [20].

These findings underscore the importance of careful implementation and user accommodation in VR interventions. Different strategies should be taken into consideration such as session duration limits, pre-intervention familiarization, and ergonomic adjustments to enhance the acceptability and usability of immersive VR in obstetrics.

## 4. Discussion

As society evolves, it is essential to adopt more innovative educational tools like VR to better engage these young learners. By embracing new ways of studying and deepening medical knowledge, VR can enhance educational experiences, promote continuous learning, and better prepare healthcare professionals for the challenges of modern obstetric care.

The application of VR in medical sciences has gained significant momentum, with a growing interest observed in the field of obstetrics. A literature review from 2002 highlighted the theoretical benefits and potential of VR in healthcare, laying the groundwork for future research [34]. Over the years, this theoretical framework has evolved into practical interventions, with VR being increasingly utilized not only to enhance medical skills but also to reduce pain and anxiety in patients. A 2021 scoping review underscored the applicability of VR for obstetrical patients, particularly in pain and anxiety reduction and exercise training, while emphasizing the need for studies with larger sample sizes to establish definitive conclusions regarding its benefits for this patient population [35].

Similarly, a recent meta-analysis on the use of VR for pain management during labor reported findings consistent with those observed in this review. However, the included studies were limited by smaller sample sizes [36]. In this systematic review, we prioritized the inclusion of RCTs with larger sample sizes to enhance the reliability of our findings and reduce the overall risk of bias. One exception was made for a study with fewer than 40 participants, given its focus on specialized medical intervention and the limited research available in this specific area. This approach was intended to provide a more robust evaluation of VR’s role in obstetrics, supporting its integration into both patient care and medical education.

### Limitations

In our analysis of the studies included in this review, we encountered several limitations that are worth mentioning to offer a balanced perspective.

Firstly, we need to mention the heterogeneity present in the studies. The diverse population studied included medical students, medical specialists and nurses to medical interventions using VR may introduce variability of generalization of the results. Also, the difference in virtual reality technology can lead to challenges in comparing effectiveness.

Variations in methodology and the difficult task of blinding participants in studies using VR for pain management during labor or other obstetric procedures, because the participants are aware of their exposure to the immersive VR environment, should be taken into consideration when interpreting the results.

Another limitation is the exclusion of studies where full texts were not accessible. This might have led to the omission of valuable data, contributing to potential publication bias.

While VR offers an immersive and controlled learning environment, challenges such as simulator-induced discomfort and emotional desensitization must be acknowledged and addressed through proper user guidance and debriefing. Future research should establish standardized ethical guidelines to ensure the responsible implementation of VR technology in medical education and clinical practice.

Despite these limitations, the valuable insights we drew can be used as a basis for future studies to build upon to further strengthen the field.

This review also identifies areas requiring further investigation, such as the long-term retention of skills acquired through VR training, the cost-effectiveness of implementing VR in medical education, and the optimal integration of VR into traditional training programs. While VR has shown effectiveness in reducing pain and anxiety during labor, outcomes for other procedures remain mixed, indicating the need for more research in this field.

## 5. Conclusions

As the findings of this systematic review demonstrate, we believe that virtual reality has the potential to improve obstetric training and patient care. As technology becomes more ingrained in our daily lives, integrating VR into medical education can better align with the learning preferences of younger generations, leading to improved healthcare delivery and better maternal and neonatal outcomes.

Overall, the integration of VR into obstetric care also offers a valuable, patient-centered approach to managing the physical and emotional challenges associated with pregnancy and childbirth.

## Figures and Tables

**Table 1:** Inclusion and exclusion criteria.

Criteria	Inclusion	Exclusion
**Population**	- Healthcare professionals (obstetricians, midwives, medical students, nurses) trained using VR for obstetric procedures. - Obstetrical patients experiencing labor and delivery.	- Studies not focused on obstetric populations or obstetrical procedures.
**Intervention**	- Use of VR as a training tool for obstetric procedures (e.g., cesarean sections, labor management, delivery techniques, patient care). - VR interventions to reduce pain, anxiety, or improve patient satisfaction during labor/delivery.	- Studies using VR for non-obstetric purposes. Studies focusing solely on the technical aspects of VR without examining outcomes like pain or anxiety.
**Comparison**	- Studies comparing VR training to traditional methods like simulation-based training, hands-on practice, or lectures.	- Studies lacking a comparison between VR and traditional training or interventions.
**Outcomes**	- Healthcare professionals’ skills, knowledge, confidence, or patient care quality following VR training. - Patient outcomes such as pain, anxiety, or satisfaction during obstetrical procedures and labor.	- Studies not investigating training effectiveness or patient care improvements.
**Study Design**	- Randomized controlled trials (RCTs); experimental studies	- Studies with designs outside the specified categories (e.g., purely observational studies without intervention).
**Language**	- Articles published in English.	- Non-English articles.
**Publication Date**	- Studies published between 1 January 2002 and 12 December 2024	- Studies published outside the specified timeframe.
**Full-Text Availability**	- Studies with accessible full texts.	- Studies with only abstracts available and no accessible full texts, despite retrieval efforts.

**Table 2:** Risk of bias assessment.

Study	Randomization Process	Deviations from Intended Interventions	Missing Data	Measurement of Outcome	Selection of Reported Results	Overall Bias
Kleiner et al. [15]	Low Risk	Low Risk	Low Risk	Low Risk	Low Risk	Low Risk
Dunlop et al. [16]	Low Risk	Some Concerns	Low Risk	Low Risk	Low Risk	Some Concerns
Falcone et al. [17]	Low Risk	Low Risk	Low Risk	Low Risk	Low Risk	Low Risk
Kim et al. [18]	Low Risk	Low Risk	Low Risk	Low Risk	Low Risk	Low Risk
Estrella-Juarez et al. [19]	Low Risk	Low Risk	Low Risk	Some Concerns	Low Risk	Some Concerns
Mohammadi et al. [20]	Low Risk	Low Risk	Low Risk	Low Risk	Low Risk	Low Risk
Orhan et al. [21]	Low Risk	Some Concerns	Low Risk	Some Concerns	Low Risk	Some Concerns
Ayca Solt Kirca et al. [22]	Low Risk	Some Concerns	Low Risk	Some Concerns	Low Risk	Some Concerns
Jeong et al. [23]	Low Risk	Some Concerns	Low Risk	Some Concerns	Low Risk	Some Concerns
Carus et al. [24]	Low Risk	Some Concerns	Low Risk	Low Risk	Low Risk	Some Concerns
Almedhesh et al. [25]	Low Risk	Low Risk	Low Risk	Low Risk	Low Risk	Low Risk
Ebrahimian et al. [26]	Low Risk	Some Concerns	Low Risk	Low Risk	Low Risk	Some Concerns
Kane et al. [27]	Low Risk	Low Risk	Low Risk	Low Risk	Low Risk	Low Risk
Melcer et al. [28]	Low Risk	Low Risk	Low Risk	Low Risk	Low Risk	Low Risk
Akin et al. [29]	Low Risk	Low Risk	Low Risk	Some Concerns	Low Risk	Some Concerns
Gür et al. [30]	Low risk	Some Concerns	Low Risk	Some Concerns	Low Risk	Some Concerns
Smith et al. [31]	Low Risk	Some Concerns	Low Risk	Low Risk	Low Risk	Some Concerns
Noben et al. [32]	Low Risk	Some Concerns	Low Risk	Low Risk	Low Risk	Some Concerns

**Table 3:** VR Studies Focused on Patients.

Authors	Year	Objectives	Type of VR Used	Participants (*n*)	Conclusions
Kleiner et al. [15]	2024	Investigate VR during extra-amniotic balloon insertion for pain and anxiety.	Immersive	132 women undergoing labor induction	VR significantly reduced pain and anxiety, with high satisfaction levels reported.
Estrella-Juarez et al. [19]	2023	Evaluate VR and music therapy on maternal and fetal physiologic parameters.	Immersive	343 pregnant women in 3rd trimester	VR and music therapy reduced maternal anxiety, blood pressure, and heart rate, showing positive fetal outcomes.
Mohammadi et al. [20]	2023	Investigate VR’s effect on fear of pain and labor pain intensity.	Immersive	130 pregnant women in labor	VR significantly reduced labor pain intensity and fear of childbirth.
Orhan et al. [21]	2023	Assess VR glasses for pain relief and satisfaction during episiotomy repair.	Immersive	50 women undergoing episiotomy	VR glasses reduced pain and increased satisfaction during episiotomy repair.
Ayca Solt Kirca et al. [22]	2023	Investigate VR glasses for pain and anxiety during episiotomy repair.	Immersive	120 primiparous women	VR glasses significantly reduced pain and anxiety during the procedure.
Carus et al. [24]	2022	Investigate immersive VR for childbirth experience and pain relief.	Immersive	42 women in labor	VR improved patient satisfaction and reduced pain scores during childbirth.
Almedhesh et al. [25]	2022	Assess VR for anxiety, stress, and hemodynamic parameters during cesarean.	Immersive	351 women undergoing cesarean	VR reduced anxiety, stress, and improved maternal satisfaction during cesarean section.
Ebrahimian et al. [26]	2022	Compare the effectiveness of VR and chewing mint gum for labor pain.	Immersive	93 women in labor	Both VR and chewing gum significantly reduced labor pain and anxiety compared to the control group.
Melcer et al. [28]	2021	Assess analgesic efficacy of VR for acute pain during amniocentesis.	Immersive	60 women undergoing amniocentesis	VR significantly reduced acute pain levels during amniocentesis.
Akin et al. [29]	2021	Investigate VR fetal images during labor and its impact on pain and anxiety.	Immersive	100 laboring women	VR reduced pain, anxiety, and positively influenced the birth experience.
Gür et al. [30]	2020	Evaluate the impact of cognitive-behavioral techniques combined with VR on birth pain.	Immersive	273 laboring women	Showed significant pain reduction in intervention groups using newborn photographs and classical music.
Smith et al. [31]	2020	Assess VR for analgesia during external cephalic version.	Immersive	50 women undergoing ECV	VR was feasible but did not show significant improvement in pain compared to the control group.
Noben et al. [32]	2019	Evaluate VR for improving preoperative information before cesarean delivery.	Non-Immersive	97 pregnant women scheduled for CS	85% felt better prepared for the procedure, but VR did not significantly reduce preoperative anxiety.

**Table 4:** VR studies focused on medical training.

Authors	Year	Objectives	Type of VR Used	Participants (*n*)	Conclusions
Dunlop et al. [16]	2024	Assess the effectiveness of VR training in knowledge of Postpartum Hemorrhage (PPH).	Non-immersive	34 medical students	VR enhances students’ knowledge and confidence in the topic covered.
Falcone et al. [17]	2024	Assess effectiveness of VR for shoulder dystocia management training.	Immersive	61 medical and nursing students, residents, and attending physicians	VR enhanced psychomotor skills and confidence in managing shoulder dystocia.
Kim et al. [18]	2024	Evaluate VR training in cesarean section (CS) and premature rupture of membranes (PROM) management.	Immersive	105 trainees (medical residents, students)	VR significantly improved confidence and knowledge in performing CS and managing PROM.
Jeong et al. [23]	2024	Evaluate VR training in maternity care.	Immersive	59 junior nursing students	VR improved knowledge and confidence in vaginal delivery.
Kane et al. [27]	2022	Evaluate VR learning environment’s effect on obstetric medical student teaching.	Immersive	69 medical students	VR improved student knowledge and confidence in obstetric scenarios.

**Table 5:** VR intervention and evaluation methods used in studies for medical training.

Study	VR Intervention	Comparison Group	Knowledge Evaluation Method	Other Measured Outcomes
Dunlop et al. (2024) [16]	VR Learning Environment (VRLE) tutorial on postpartum PPH	PowerPoint tutorial on the same topic	Pre-learning and post-learning surveys; assessment of PPH balloon insertion technique on a model pelvis	Confidence levels, time taken to complete the task, technique assessment, satisfaction, side effects of VR
Falcone et al. (2024) [17]	360° VR video of shoulder dystocia (SD) management	Theoretical briefing on SD management	HuFSHI and HELP-RER score assessments at baseline and after manikin-based training	Diagnosis-to-delivery time, task-load index (TLX), crossover study design
Kim et al. (2024) [18]	VR simulation training for cesarean section and PROM management	Video presentation of the clinical scenario and CS procedure	Pre- and post-training questionnaire on VR experience, CS and PROM management	Mini-test quiz, confidence levels
Jeong et al. (2024) [23]	VR simulation class on normal vaginal delivery care (IVR headset)	Traditional simulation training using SimMom full-body birthing simulator	Pre-test and post-test using a 20-item knowledge scale on normal vaginal delivery care	Satisfaction with simulation education, self-efficacy, confidence
Kane et al. (2022) [27]	VRLE exploring fetal lie and presentation during the third trimester	Self-directed study of 2D images of fetal lie and presentation with printed descriptions	Correct determination of fetal lie and presentation on an obstetric model	Task completion time, student satisfaction and self-confidence in learning

**Table 6:** Studies investigating pain relief.

Study	Outcome	VR Effect	Key Observations
Kleiner et al., 2024 [15]	Pain Relief	Moderate reduction in pain scores	Effective for labor induction with extra-amniotic balloon insertion.
Estrella-Juarez et al., 2023 [19]	Pain Relief	Combined VR and music reduced pain	Improved maternal and fetal outcomes during labor.
Mohammadi et al., 2023 [20]	Pain Relief	Substantial pain reduction	Demonstrated efficacy in active labor pain management.
Orhan et al., 2023 [21]	Pain Relief	Significant pain reduction	Effective for episiotomy repair; also improved satisfaction.
Ayca Solt Kirca et al., 2023 [22]	Pain Relief	Substantial reduction in pain	Focused on primiparous women during episiotomy repair.
Carus et al., 2022 [24]	Pain Relief	Moderate reduction in labor pain	Enhanced patient satisfaction and childbirth experience.
Ebrahimian et al., 2022 [26]	Pain Relief	Significant reduction in labor pain	VR and chewing gum were both impactful in lowering pain levels during labor.
Akin et al., 2021 [29]	Pain Relief	Large reduction in labor pain	VR was impactful in managing labor pain and positively influenced the birth experience.
Melcer et al., 2021 [28]	Pain Relief	Significant pain reduction	Demonstrated efficacy for procedural pain during amniocentesis.
Gür et al., 2020 [30]	Pain relief	Significant reduction	Showed significant pain reduction in intervention groups using newborn photographs and classical music
Smith et al., 2020 [31]	Pain Relief	No significant reduction	Although patients going through external cephalic version did not experience lower pain levels during the procedure, 80% of the participants would recommend VR to others.

**Table 7:** Studies investigating anxiety reduction.

Study	Outcome	VR Effect	Key Observations
Estrella-Juarez et al., 2023 [19]	Anxiety Reduction	Substantial reduction in anxiety	Combined VR and music therapy was highly effective during labor.
Mohammadi et al., 2023 [20]	Anxiety Reduction	Moderate reduction in childbirth anxiety	Highlighted VR’s role in reducing fear of childbirth.
Ebrahimian et al., 2022 [26]	Anxiety Reduction	Significant reduction in labor anxiety	VR and chewing gum were both impactful in lowering anxiety levels during labor.
Almedhesh et al., 2022 [25]	Anxiety Reduction	Significant anxiety reduction	Effective during caesarean sections; also improved maternal satisfaction.
Melcer et al., 2021 [28]	Anxiety reduction	No significant anxiety reduction	No statistically significant anxiety reduction was observed during amniocentesis.
Noben et al., 2019 [32]	Anxiety Reduction	Preoperative anxiety reduction	VR improved preparedness for caesarean but did not show strong effects on overall anxiety levels.

**Table 8:** Studies investigating training outcomes.

Study	Outcome	VR Effect	Key Observations
Dunlop et al., 2024 [16]	Knowledge Improvement	Substantial improvement	Focused on postpartum haemorrhage management.
Falcone et al., 2024 [17]	Psychomotor Skills	Substantial improvement in skills and confidence	VR significantly enhanced shoulder dystocia training.
Kim et al., 2024 [18]	Knowledge Retention	Improved confidence and technical skills	Focused on caesarean section and PROM management training.
Jeong et al., 2024 [23]	Knowledge Retention	Increased knowledge retention and confidence	Effective for normal vaginal birth scenarios
Kane et al., 2022 [27]	Student Knowledge	Substantial knowledge and confidence gain	Evaluated VR as an educational tool in obstetrics.

**Table 9:** Types of VR used.

Type of VR	*N* Studies
Immersive VR (Headsets)	16 [15,17,18,19,20,21,22,23,24,25,26,27,28,29,30,31]
Non-Immersive VR (2D)	2 [16,32]

## Data Availability

No new data were created or analyzed in this study. Data sharing is not applicable to this article.
